# Metaphor in embodied cognition is more than just combining two related concepts: a comment on Wilson and Golonka (2013)

**DOI:** 10.3389/fpsyg.2013.00201

**Published:** 2013-04-22

**Authors:** Jens H. Hellmann, Gerald Echterhoff, Deborah F. Thoben

**Affiliations:** ^1^Department of Psychology, Center of Higher Education, University of MünsterMünster, Germany; ^2^Social Psychology, University of MünsterMünster, Germany; ^3^Criminological Research Institute of Lower SaxonyHanover, Germany

In their recent article on embodied cognition, Wilson and Golonka (2013) also discuss research on conceptual metaphors like “power is up” or “the future is forward” to exemplify common approaches to embodied cognition. Metaphors are particularly interesting for embodied cognition research because they can map concrete, bodily experiences onto abstract concepts (Lakoff and Johnson, 1980). To be sure, one could take issue with the selection of the two specific studies (Miles et al., 2010; Eerland et al., 2011). Only the latter study (Miles et al., 2010), which examined the relation between mental time travel and bodily posture, involves a conceptual metaphor, whereas the former study (Eerland et al., 2011) invokes the notion of a mental number line rather than any conceptual metaphor and indeed demonstrates that sometimes cognitions and bodily postures go together. There are many other interesting demonstrations that are consistent with the notion that conceptual metaphors inform and shape thinking (for a review, see Landau et al., 2010).

More importantly, we believe that to appreciate the joint operation of bodily experience and metaphors one needs to take into account metaphors that are actually articulated and encountered in linguistic practice. Conceptual metaphors represent general mappings that are assumed to organize and facilitate thought and judgments. Often, they are inferred and formulated by researchers, but many of them are not, or only exceptionally, used in non-scientific, everyday speech, and discourse. However, to understand how physically rooted language helps people perform tasks such as judgments or decisions in the real world, one needs to study metaphoric devices that are commonly used in language communities, that is, idiomatic or conventional metaphors like “alcohol is a crutch” or “revenge is sweet” (Holland, 1982; Burbules et al., 1989). The source concept of an idiomatic metaphor often represents a concrete bodily or physical state, whereas the target concept is relatively abstract. Idiomatic metaphors are relevant to embodied cognition precisely because they involve the concurrent activation of a bodily sensation and an abstract cognitive concept, which jointly guide people's cognition.

Research on embodied cognition has focused on only one type of metaphor, that is, conceptual metaphors. But idiomatic metaphors differ in several key aspects from conceptual metaphors, and these differences have important implications for theorizing on embodied cognition (Hellmann et al., in press). In the following, we will briefly explain the differences between conceptual and idiomatic metaphors and then point to implications of idiomatic metaphors for embodied cognition research and theorizing.

Conceptual metaphors combine two entities that intuitively fit together like weight and importance (Jostmann et al., 2009; Schneider et al., 2011). However, they are typically not used in speech and everyday language as frequently as idiomatic metaphors. Conceptual metaphors primarily represent inferences that guide individuals' cognitions. This effect emerges because one concept often spontaneously activates another, intuitively similar, concept. However, the source concept is not necessarily a concrete physical experience, and the target concept does not necessarily represent a rather abstract domain (see Schneider et al., 2011).

For an idiomatic metaphor to guide individuals' cognitions toward relevant judgments, there are more cognitive limitations than for a conceptual metaphor: The associations between the two concepts of the metaphor are less strong, less intuitive, and less stable in idiomatic metaphors. There is a more limited applicability of idiomatic metaphors than of conceptual metaphors. While conceptual metaphors often work both ways, that is, they are bi-directional, idiomatic metaphors operate one-way, that is, uni-directionally. Additionally, when an idiomatic metaphor is reversed it loses its original and genuine sense (Glucksberg et al., 1997; Landau et al., 2010).

Because the associations are less stable and less intuitive in an idiomatic metaphor, it requires the concurrent activation of both of its concepts (see Hellmann et al., in press). Source and target concept have to be specifically activated to in a given situation to provide a new understanding of the target concept and shape individuals' thinking and judgments. Evidence this for this constraint is provided by our own recent research (Hellmann et al., in press). In two experiments, we investigated whether the sensation of sweet taste informs judgments of harmful acts via indirect activation of the idiomatic metaphor “Revenge is sweet.” We found in one study that only after priming with the concept revenge, but not after priming with the similar concept schadenfreude, a concurrent sweet (*vs*. fresh) taste led to more lenient judgments. Hence, a physical state per se is not sufficient for effects of idiomatic metaphors on cognition.

The differences between the two types of metaphor have important implications for the understanding of embodied cognition. Conceptual metaphors can affect cognition already when only one of the pertinent concepts (source or target concept) is activated because the other concept will be automatically co-activated (see Barsalou, 2003). As our research suggests, idiomatic bodily metaphors can affect cognition only when both source concept and target concept are sufficiently activated such that the source concept can actually be mapped onto the target concept in a given situation.

To conclude, if one wants to understand the role of linguistic devices like metaphors in embodied cognition, one should take into account and appreciate the role of idiomatic metaphors. Following the terminology suggested by Wilson and Golonka (2013), such linguistic devices often serve as resources for the performance of real-world tasks (also see IJzerman and Koole, 2011). Importantly, to the extent that idiomatic metaphors are distinctive means for integrating bodily experiences into thinking, they certainly deserve attention by researchers interested in embodied cognition.
